# Carbapenem treatment options for metallo-beta-lactamase: drug screening and dose optimization of meropenem-based combinations against NDM- or IMP-producing *Klebsiella pneumoniae*

**DOI:** 10.3389/fmicb.2025.1490372

**Published:** 2025-04-15

**Authors:** Haoyue Che, Bo Hu, Xiaoming Cui, Jiyong Yang, Jinru Zeng, Liuhan Dong, Rui Wang, Yun Cai

**Affiliations:** ^1^Department of Pharmacy, Center of Medicine Clinical Research, Medical Supplies Center, PLA General Hospital, Beijing, China; ^2^Graduate School of the PLA General Hospital, Beijing, China; ^3^Department of Health Medicine, The Third Medical Centre, Chinese PLA General Hospital, Beijing, China; ^4^Clinical Laboratory, PLA General Hospital, Beijing, China

**Keywords:** meropenem, fosfomycin, colistin (CST), *in vitro* PK/PD model, MBL-KP

## Abstract

**Background:**

The global dissemination of carbapenemase-producing *Klebsiella pneumoniae* (CPKP) has been occurring at an alarming pace, especially for the metallo-β-lactamase (MBL) group. Current clinical data suggest that carbapenem is still irreplaceable in terms of safety and efficacy. For MBL-producing *Klebsiella pneumoniae* (MBL-KP), how to use carbapenem judiciously in the context of advocating carbapenem-sparing strategies remains largely undetermined.

**Methods:**

Four strains carrying different MBLs (two IMPs and two NDMs) were collected. The genome sequence, drug resistance phenotype, and synergistic effect of different meropenem-based antimicrobial combinations were tested. Dynamics *in vitro* pharmacokinetic/pharmacodynamic (PK/PD) model was used to optimize the dosage. The selected combination regimens were verified in a murine model of peritoneal sepsis.

**Results:**

Meropenem in combination with fosfomycin or colistin was effective against MBL-KP strains. Meropenem in combination with colistin had a better bactericidal effect compared with fosfomycin, while such a combination was prone to cause colistin-dependent heterogeneous resistance, especially for IMP-KP. For NDM-KP extremely resistant to meropenem, ultra-high dose up to 2.75 g q6h meropenem in combination with fosfomycin or colistin was needed. For IMP-KP, high-dose meropenem monotherapy (2 g q8h) or low-dose meropenem (1 g q8h) in combination with fosfomycin were both feasible.

**Conclusion:**

Meropenem in combination with fosfomycin or colistin was an effective choice for MBL-KP. The combination regimen and dosage optimization should be assessed based on not only the type of enzyme but also the specific value of minimum inhibitory concentration (MIC).

## 1 Introduction

As one of the Enterobacteriaceae, *Klebsiella pneumoniae* (KP) has gradually become an important pathogen of hospital infections (Martin and Bachman, 2018). The 2024 WHO Bacterial Priority Pathogens List showed that carbapenem-resistant Enterobacterales (CRE) continue to be at the top ranking in terms of the need for new therapy. These pathogens pose the highest estimated burden among all multi-drug resistance Gram-negative bacteria due to their widespread prevalence and resistance (World Health Organization, 2024). In 2024, the resistance rates of KP in China to imipenem and meropenem were 18.2 and 18.6% respectively, marking 3 consecutive years of continuous increase at a high detection level (CHINET, 2024). The literature search on carbapenem-resistant infections in neonates and children in Latin America revealed that the most frequently reported isolates were KP, with a mortality rates ranging from 13 to 52.6% (Shanks and Grandjean, 2025). All above emphasized the need to strengthen the management and monitoring CPKP and its associated antibiotics. Transmissible carbapenem-resistance in KP has been recognized for the past two decades, whereas the global spread of CRKP is a more recent issue and once it initiates, the infection occurs at an alarming pace (Logan and Weinstein, 2017).

The current carbapenemase inhibitors mainly target KP carbapenemases (KPC) and oxacillinase (OXA), effective treatment options for MBLs remain elusive (Pitout et al., 2015). CPACKLE-2 research shows that global CRKP epidemics have important regional differences in bacterial characteristics, the effect of which on individualized administration is unclear (Wang et al., 2021). Studies have shown that for KP strains characterized by carbapenem “low-level resistance” (MIC of 16-32 mg/L), combinations containing a carbapenem exert the best results in decreasing hospital mortality (Paul et al., 2014). The role of carbapenem in the treatment of CRKP has long been debated. On the one hand, clinical practitioners are encouraged to use carbapenem as sparingly as possible in order to preserve their efficacy. On the other hand, for CRKP, when the effect of alternative drugs is unsatisfactory or difficult to obtain, it is necessary to make appropriate dose adjustments and a combination of carbapenem to improve the microbiological outcomes and limit the occurrence of drug resistance. How to use carbapenem judiciously for MBL-KP in the context of advocating carbapenem-sparing strategies remains largely unclear.

In the present study, a series of *in vivo* and *in vitro* experiments were conducted to explore the appropriate meropenem-based combinations against clinically isolated MBL-KP, in which the resistance genotype, phenotype, and MIC values were all clarified.

## 2 Materials and methods

### 2.1 Bacterial isolates

Four clinical KP strains carrying MBL were isolated from patients in the Chinese People’s Liberation Army (PLA) General Hospital. Two strains (KP-N1 and KP-N2) produced NDM1, and the other two strains (KP-I1 and KP-I2) produced IMP.

### 2.2 Strain characteristics

#### 2.2.1 Whole-genome sequencing

To identify the genomic resistance, the above-mentioned four strains were subjected to WGS. Multilocus sequence typing (MLST) was determined using the MLST 1.8 server^1^. The ResFinder 2.1 tool of the Center for Genomic Epidemiology (CGE) website^2^ was used to screen the common resistance genotypes of four strains and obtain the resistance genes they carry.

#### 2.2.2 Real-time reverse-transcription polymerase chain reaction

The gene expression at the mRNA level was determined by RT-PCR. The expressions of *bla*_*NDM*_ and *bla*_*IMP*_ and the relative expressions of *acrB*, *ompk35*, and *ompk36* were determined. The *rpoB* gene was selected as the housekeeping gene, the KP ATCC700603 was used as the reference strain for *acrB*, and the KP ATCC13883 was used as the reference strain for *ompk35* and *ompk36* (expression level = 1). The relative expressions of the target genes were calculated using the 2^–ΔΔ^*^CT^* method. All primer sequences were listed in Supplementary Table 1.

### 2.3 Antimicrobial agents

Meropenem (Macklin, Shanghai, China) was tested alone and in combination with the following antimicrobials: ertapenem (MedChemExpress, New Jersey, United States), colistin sulfate (MedChemExpress, New Jersey, United States), amikacin (National Institutes for Food and Drug Control, Beijing, China), fosfomycin sodium for injection (NORTHAST PHARM, Shanghai, China), and rifampin (National Institutes for Food and Drug Control, Beijing, China).

### 2.4 Synergy testing and combination regimen screening

MIC was tested by the microdilution broth method according to the guidelines of the Clinical and Laboratory Standards Institute (CLSI) (Clinical and Laboratory Standards Institute, 2021). The concentration ranges of the experimental antibiotics were: meropenem (0.25-128 μg/mL), colistin sulfate (0.125-8 μg/mL), amikacin (0.25-128 μg/mL), fosfomycin sodium for injection (4-128 μg/mL), rifampin (1-64 μg/mL). The fractional inhibitory concentration index (FICI) was calculated, synergy as FICI of ≤ 0.5, indifference as 4 ≥ FICI > 0.5, and antagonism as FICI of > 4 (Odds, 2003). The combination regimen with synergistic effect was screened out for further study.

### 2.5 *In vitro* dose optimization with PK/PD model experiments

The *in vitro* PK/PD model (PASS-402W System, Japan) was used to conduct dynamic time-kill experiments of the combination regimens screened out earlier. The system diagram was illustrated in Supplementary Figure 1. According to the PK characteristics of different drugs, changes in concentrations of both antibiotics with clinical doses were simulated by the *in vitro* PK/PD model. The initial bacterial concentration in the central compartment was about 10^7^ CFU/mL.

#### 2.5.1 Dynamic time-kill experiments

Time-kill analyses were performed on the four isolates. According to the sampling time set, the bacterial solution was taken from the central compartment to count the bacterial colony, and the time sterilization curve under different administration schemes was drawn.

#### 2.5.2 Population analysis profile

For the combination regimen including colistin, the bacterial solutions of 0, 24, and 48 h were taken for PAP. The colony was incubated overnight on Mueller-Hinton Agar (MHA) containing different concentrations of colistin (0.25-64 mg/L), and the PAP curve was obtained.

### 2.6 Efficacy verification *in vivo* using a murine model of peritoneal sepsis

Infected mice were randomly divided into the controls groups (untreated), monotherapy groups, and combination groups. The antimicrobial dosages were selected based on the PK/PD data in previous studies (Lepak et al., 2017, Oshima et al., 2017, Papp-Wallace et al., 2019). The survived mice were sacrificed through inhalation of isoflurane (Veteasy, Shenzhen, China) at 24 h. Samples were extracted and processed immediately. At 24 h, the blood sample was taken through eyeball extraction, spleen samples were aseptically extracted, weighed, and homogenized in sterile saline, and quantitative cultures were determined by MHA plates. In the experiment containing colistin, all samples were additionally coated with four times colistin at the MIC concentration for quantification to verify the formation of heterogeneous drug resistance in animals.

## 3 Results

### 3.1 Strain characteristics

**FIGURE 1:**
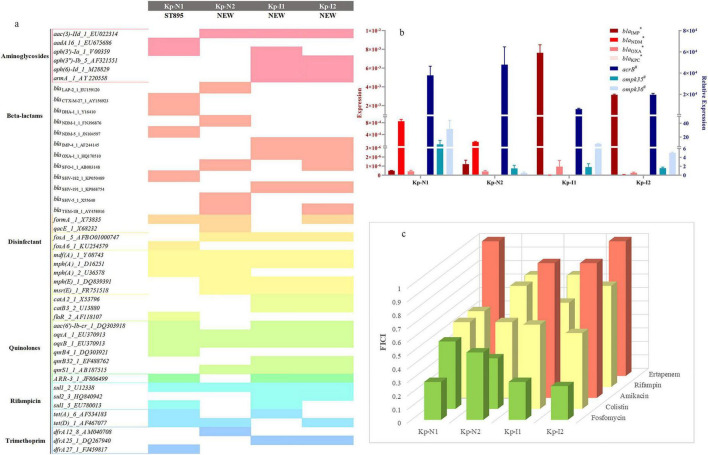
Strains characteristics and combination regimen screen. **(a)** Sequence type and resistance genotype of four MBL-KP strains. The color squares indicate the presence of each gene. **(b)** RT-PCR results of *bla*_NDM_, *bla*_IMP_, *bla*_OXA_, *bla*_KPC_, *acrB*, and *ompk*. *Gene results are shown as an expression with a quantity ratio of *Bla* to that of *rrsE*.^#^Gene results are shown as a relative expression with the expression compared with ATCC13883 and ATCC700603, repectively. **(c)** Synergistic effect of meropenem and other antimicrobials using the chequerboard assay. The x-axis represents different types of strains, the y-axis represents different antimicrobials, and the prism height represents the FICI value. Green, yellow, and red prism represent that FICI ≤ 0.5 with synergistic effect, 0.5 < FICI ≤ 1 with partial synergy effect, and FICI > 1 with indifferent or antagonism effect, respectively.

Figure 1a shows the sequence type and resistance genotype. Except for KP-N1, which belonged to ST895, all the other strains belonged to unclassified new ST types. The expressions of enzyme-producing genes and efflux pump genes of NDM-KP were lower compared with IMP-KP, and there was no obvious loss of membrane porin genes. Compared with KP-I1, KP-I2 had significantly higher expressions of efflux pump genes and lower expressions of membrane porin genes, while the expressions of enzyme-producing genes were relatively low (Table 1 and Figure 1b).

**TABLE 1:** Characteristics of MICs, β-lactamase, efflux pump and porin of four strains.

strains	MIC (mg/L)							acrB	ompK		IMP	NDM
	MEM	FOS	CST	AMIK	RFP	ETP	CAZ/AVI	Gene dosage^a^	Gene dosage (ompK35)^b^	Gene dosage (ompK36)^b^	Gene dosage^c^	Gene dosage^c^
Kp-N1	64	16	1	1	32	≥16	≥128	6132.20 ± 756.83	1.83 ± 0.76	10.03 ± 0.46	−	0.001 ± 6.08e-005
Kp-N2	16	32	1	0.5	32	≥16	≥128	19586.70 ± 1354.50	1.59 ± 0.22	5.03 ± 0.15	−	0.00015 ± 1.9e-005
Kp-I1	4	32	1	≥256	≥128	8	≥128	37851.52 ± 8672.20	9.42 ± 5.70	31.52 ± 12.86	0.09 ± 0.0010	−
Kp-I2	16	128	0.5	≥256	≥128	≥16	≥128	47931.54 ± 16739.22	1.52 ± 0.71	0.50 ± 0.22	0.0030 ± 7.05e-005	−

MEM, meropenem; FOS, fosfomycin; CST, colistin; AMIK, amikacin; RFP, rifampin; ETP, ertapenem; CAZ/AVI, ceftazidime/avibactam. RT-PCR results of blaNDM, blaIMP, blaOXA, blaKPC, acrB and ompk.

^a^Genes results are shown as expression with a quantity ratio of *Bla* to that of *rrsE*.

^b^Gene results are shown as relative expression with the expresson compared to ATCC13883.

^c^Gene results are shown as relative expression with the expresson compared to ATCC700603. − Expression level was lower than e-006.

Table 1 show the resistance phenotype of the strains. The MIC of meropenem was 64 and 16 mg/L for NDM-KP, and 4 and 16 mg/L for IMP-KP. The MIC of fosfomycin was 16 and 32 mg/L for NDM-KP, and 32 and 128 mg/L for IMP-KP. All four strains were sensitive to colistin. Additional clinical information is provided in Supplementary Table 1.

### 3.2 FICI

The checkerboard assay showed that the most significant synergistic effect was mainly observed from regimens of meropenem in combination with colistin or fosfomycin, which were selected for the subsequent *in vitro* and *in vivo* studies (Figure 1c).

### 3.3 *In vitro* PK/PD model experiments

According to previous clinical research, the recommended meropenem doses are 0.5, 1, and 2 g q8h (Gu et al., 2016), and the highest meropenem is added to 2.75 g q6h for severe CRKP infections (Choi and Ko, 2015). Two-compartment model was used to describe the PK characteristics of meropenem, and the PK parameters are shown in Supplementary Table 3 (Bian et al., 2019), a prolonged infusion time of 3 h was adopted in the present study (Andrade et al., 2014).

Fosfomycin is safe in the clinical application at a dose of 8 g q8h (Magiorakos et al., 2012), and Supplementary Table 3 shows the PK parameters of the single-compartment model.

Colistin has PK characteristics of the non-compartmental model (Tsuji et al., 2019) and the recommended steady-state mean plasma concentration (Css) of clinical colistin is 2 mg/L, which is the maximum tolerable dose for treatment. The simulated central compartment steady-state concentration of colistin is maintained at or below 2 mg/L (Andrade et al., 2014).

#### 3.3.1 Time-kill curves, log change at 24 h (ΔlgCFU_24_), and area under the time-killing curves

**FIGURE 2:**
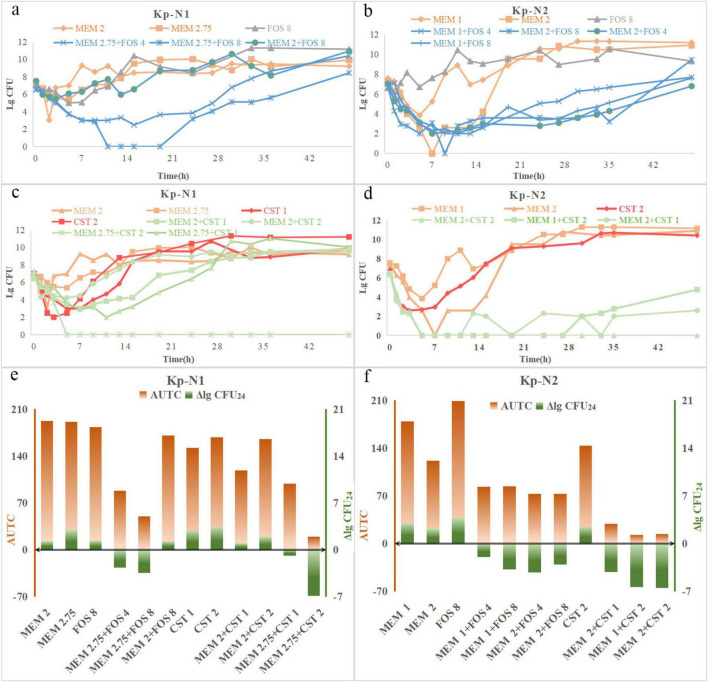
Time-course profiles of **(a)** Kp-N1, **(b)** Kp-N2 colony counts for meropenem, fosfomycin, and their combinations, **(c)** Kp-N1, **(d)** Kp-N1 colony counts for meropenem, colistin, and theirs combinations. ΔlgCFU_24_ and AUTC of **(e)** Kp-N1, **(f)** Kp-N2 for different regimens. MEM 1, meropenem 1 g q8h; MEM 2, meropenem 2 g q8h; MEM 2.75, meropenem 2.75 g q6h; FOS 4, fosfomycin 4 g q8h; FOS 8, fosfomycin 8 g q8h; CST 1, colistin Css = 1 mg/L; CST 2, colistin Css = 2 mg/L.

For KP-N1, the bactericidal effect was observed only when the dose of meropenem was increased to 2.75 g q6h and combined with other antimicrobials (Figures 2a,c). For KP-N2, all the monotherapy regimes were ineffective, the combination regimes showed a similar synergistic bactericidal effect, no matter how the relative dose of the two components was adjusted (Figures 2b,d). ΔlgCFU_24_ and AUTC were similar with time-kill curves (Figures 2e,f).

**FIGURE 3:**
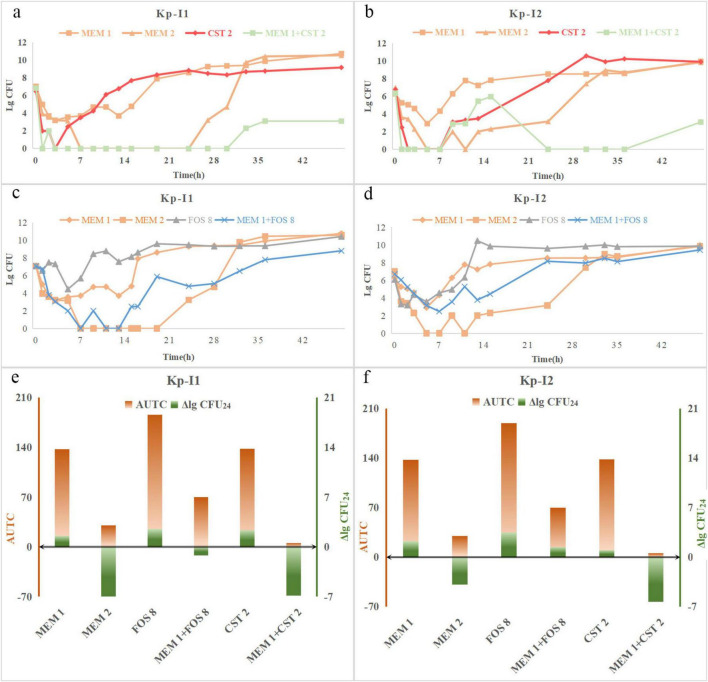
Time-course profiles of **(a)** Kp-I1, **(b)** Kp-I2 colony counts for meropenem, fosfomycin, and their combinations, **(c)** Kp-I1, **(d)** Kp-I2 colony counts for meropenem, colistin, and their combinations. ΔlgCFU_24_ and AUTC of **(e)** Kp-I1, **(f)** Kp-N2 for different regimens. MEM 1, meropenem 1 g q8h; MEM 2, meropenem 2 g q8h; FOS 8, fosfomycin 8 g q8h; CST 2, colistin Css = 2 mg/L.

The trend of time-kill curves of two IMP-KP strains was similar (Figure 3). Among the fosfomycin combinations, the bactericidal effect of 2 g q8h meropenem was the strongest, followed by 1 g q8h meropenem + 8 g q8h fosfomycin, 1 g q8h meropenem, and 8 g q8h fosfomycin. The combination regimen of colistin showed that 2 g q8h meropenem alone had a similar effect compared with 1 g q8h meropenem + 2 mg/L colistin in the early stage, while the colonies were increased slowly after meropenem withdrawal at 24 h.

#### 3.3.2 The emergence of colistin resistance

**FIGURE 4:**
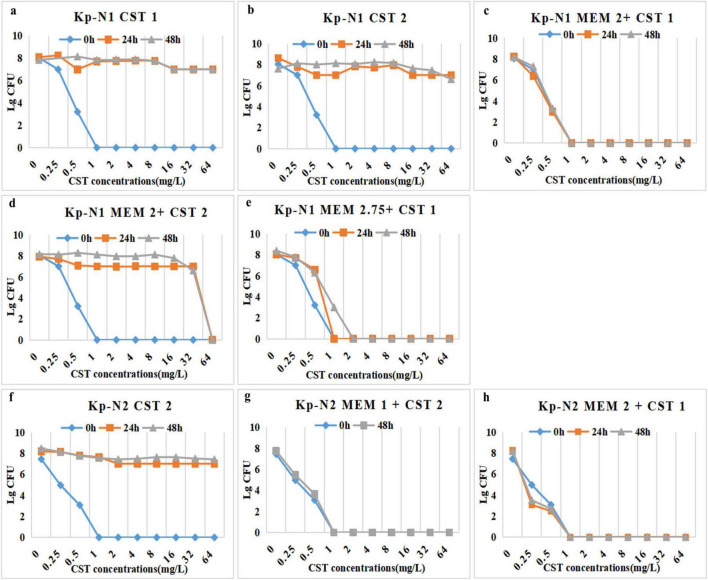
PAPs after 24 h, 48 h, and 0 h (control) of Kp-N1 for exposure to **(a)** CST 1, **(b)** CST 2, **(c)** MEM 2 + CST 1, **(d)** MEM 2 + CST 2, **(e)** MEM 2.75 + CST 1, Kp-N2 for exposure to **(f)** CST 2, **(g)** MEM 1 + CST 2, **(h)** MEM 2 + CST 1. MEM 1, meropenem 1 g q8h; MEM 2, meropenem 2 g q8h; MEM 2.75, meropenem 2.75 g q6h; CST 1, colistin Css = 1 mg/L; CST 2, colistin Css = 2 mg/L;

PAP curves showed that KP-N1 obtained the heterogeneous resistance of MIC > 64 mg/L in the colistin monotherapy regimen at 24 and 48 h (Figures 4a,b), while no heterogeneous resistance occurred in two combination regimens containing 1 mg/L colistin (Figures 4c,e). Moreover, KP-N1 obtained a heterogeneous resistance of MIC = 64 mg/L at 24 h and 48 h in the combination regimens containing 2 mg/L colistin (Figure 4d). Colistin monotherapy led to heterogeneous drug resistance of KP-N2 (Figure 4f), while the combination regimen did not (Figures 4g,h).

**FIGURE 5:**
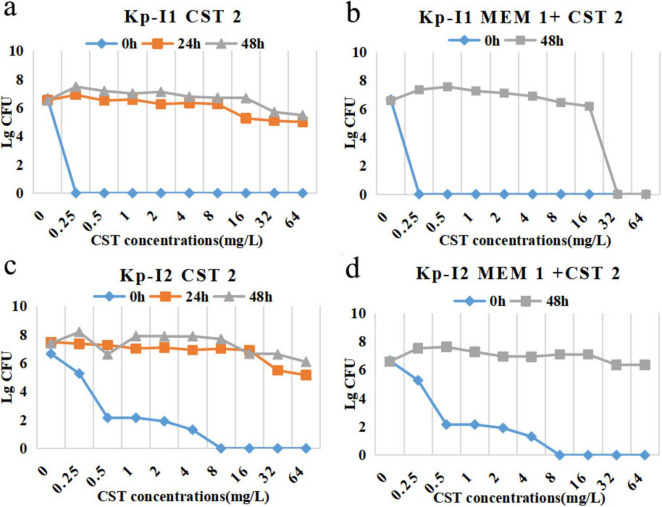
PAPs after 24 h, 48 h, and 0 h (control) of Kp-I1 for exposure to **(a)** CST 2, **(b)** MEM 1 + CST 2, Kp-I2 for exposure to **(c)** CST 2, **(d)** MEM 1 + CST 2. MEM 1, meropenem 1 g q8h; CST 2, colistin Css = 2 mg/L.

The MIC of colistin against KP-I1 was 32 mg/L at 48 h in the combination regimen (Figure 5b) compared with more than 64 mg/L of the monotherapy regimen (Figure 5a). KP-I2 had the heterogeneous resistance of MIC > 64 mg/L regardless of the monotherapy or combination therapy (Figures 5c,d).

### 3.4 *In vivo* murine model of peritoneal sepsis

KP-N1 and KP-I1 were chosen to conduct animal experiments. Table 2 shows the overall experimental grouping, dosage, mortality, and microbiological results.

**TABLE 2:** Treatment group, dose, mortality, spleen and blood bacterial enumeration in mice infected with Kp-N1 and Kp-I1.

Strains	Treatment group	Dose	Mortality (%)	Spleen			Blood		
				Number	Bacteria (Lg) Mean ± SD	Heterogeneous drug resistance rate (%)	Number	Bacteria (Lg) Mean ± SD	Heterogeneous drug resistance rate (%)
Kp-N1	Control	NS	100.00	6	6.09 ± 0.50	−	0	−	−
	MEM_low_	75 mg/kg	100.00	6	3.85 ± 0.73	−	0	−	−
	MEM_high_	150 mg/kg	16.67	6	2.67 ± 0.49	−	5	1.41 ± 1.98	−
	CST	5 mg/kg	0.00	6	3.76 ± 0.34	33.33	6	2.86 ± 0.45	83.33
	MEM_low_ + CST	75 mg/kg + 5 mg/kg	0.00	6	3.02 ± 0.16	0.00	6	1.56 ± 1.21	0.00
	MEM_high_ + CST	150 mg/kg + 5 mg/kg	0.00	6	2.10 ± 0.97	0.00	6	1.10 ± 1.21	0.00
	FOS	100 mg/kg	100.00	6	5.90 ± 0.90	−	1	3.85	−
	MEM_low_ + FOS	75 mg/kg + 100 mg/kg	66.67	5	2.98 ± 0.57	−	2	2.06 ± 2.91	−
	MEM_high_ + FOS	150 mg/kg + 100 mg/kg	83.33	6	2.34 ± 1.08	−	1	2.30	−
Kp−I1	Control	NS	100.00	6	6.99 ± 0.61	−	0	−	−
	MEM_low_	75 mg/kg	66.67	6	6.37 ± 0.45	−	2	4.55 ± 0.92	−
	MEM_high_	150 mg/kg	66.67	6	5.89 ± 1.78	−	2	5.00 ± 0.06	−
	CST	5 mg/kg	33.33	6	4.89 ± 1.20	50.00	4	4.46 ± 1.73	25.00
	MEM_low_ + CST	75 mg/kg + 5 mg/kg	0.00	6	2.81 ± 0.96	0.00	6	2.51 ± 0.50	0.00
	MEM_high_ + CST	150 mg/kg + 5 mg/kg	16.67	6	3.69 ± 1.02	0.00	5	3.09 ± 0.43	0.00
	FOS	100 mg/kg	83.33	6	3.92 ± 1.02	−	1	3.70	−
	MEM_low_ + FOS	75 mg/kg + 100 mg/kg	33.33	6	3.55 ± 0.52	−	4	3.31 ± 0.52	−
	MEM_high_ + FOS	150 mg/kg + 100 mg/kg	50.00	6	3.64 ± 1.02	−	3	3.91 ± 0.76	−

MEM_low_, low dose meropenem; MEM_high_, high dose meropenem; CST, colistin; FOS, fosfomycin; NS, normal saline. − Not available.

#### 3.4.1 Mortality and bacterial clearance

All mice in the control group without treatment died within 24 h. In mice infected with KP-N1, the mortality in the low-dose monotherapy group of fosfomycin or meropenem was 100%. All colistin-containing treatments reduced the mortality to zero. In mice infected with KP-I1, the mortality in the monotherapy group of fosfomycin or meropenem ranged from 66.7 to 88.3%. The mortality in the treatment group containing different doses of colistin ranged from 0 to 33.3% (Table 2).

**FIGURE 6:**
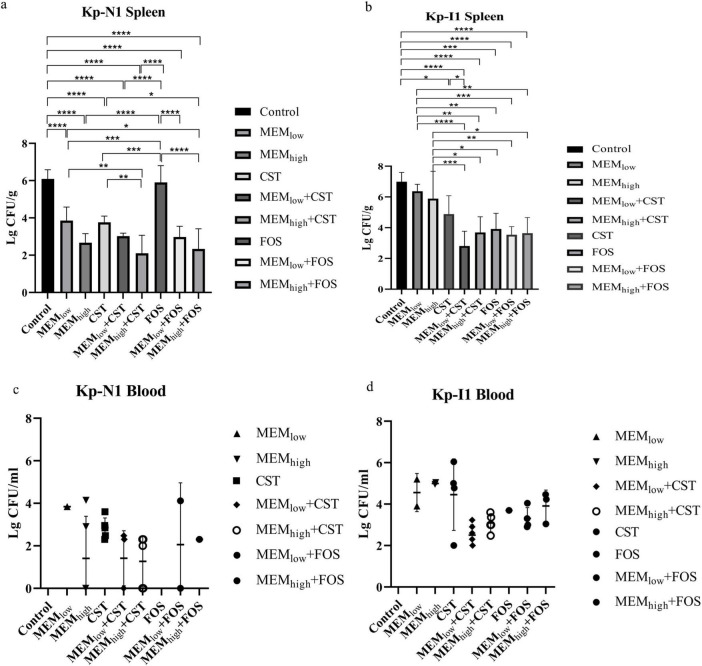
Effect of antibiotic alone or in combination on colony counts of spleen from **(a)** Kp-N1 and **(b)** Kp-I1, blood from **(c)** Kp-N1 and **(d)** Kp-I1. Results of each group are reported as mean ± SD of six mice in a, c, and survival mice at 24 h in b, d. A significance level of 0.05 was applied to all tests. *P < 0.05, **P < 0.01, ***P < 0.001, ****P < 0.0001. MEM_low_, low dose meropenem; MEM_high_, high dose meropenem; CST, colistin; FOS, fosfomycin.

Figure 6 shows that for mice infected with KP-N1, MEM_*high*_ + CST and MEM_*high*_ + FOS groups had the lowest bacterial residues. For mice infected with KP-I1, there was no significant difference among the MEM_*low*_, MEM_*high*_, and control groups. Other combination treatment groups achieved a similar bactericidal effect regardless of the meropenem dose.

#### 3.4.2 The emergence of colistin resistance

Bacterial culture of the spleen and blood samples in the colistin monotherapy group showed that both KP-N1 and KP-I2 obtained different degrees of heterogeneous drug resistance during the treatment, while the combination groups did not (Table 2).

## 4 Discussion

Although MBLs only account for 10% of carbapenemases identified since 2017 according to < AR Lab Network report > (Centres for Disease Control and Prevention, 2019), they have broad hydrolytic properties that affect most ß-lactam antibiotics and lack clinically useful inhibitors (Suay-García and Pérez-Gracia, 2019). Studies have shown that genes encoding IMP and NDM are largely plasmid-borne and can be transferred between bacterial strains, indicating that they are of particular risk in health care settings (Wu et al., 2019).

In terms of strain characteristics, we found that compared with NDM-KP, meropenem had a lower MIC for IMP-KP, which was consistent with previous research which reported most IMP-KP appeared lower MIC to meropenem (Yan et al., 2001). The MIC of two NDM-KP strains was 64 and 16 mg/L, respectively. And the expressions of porin and efflux pump genes were similar, while the expression of *bla*_NDM_ in KP-N1 was higher compared with KP-N2, which might relate to the higher meropenem resistance. Previous *in vitro* study has shown that concentration-gradient exposure of carbapenem has a significant effect on the expression of *bla*_NDM_ but not on the expressions of other resistance genes, such as the efflux pump gene in CRE (Chetri et al., 2019). Correspondingly, *in vitro* PK/PD results indicated that the dose of meropenem was critical in the regimen against KP-N1. We speculated that the dose of meropenem was related to the expression of NDM, which needs to be confirmed by further experiments. Two IMP-KP strains were resistant to meropenem at lower MIC values of 4 and 16 mg/L, respectively. At mRNA levels, the expression of *bla*_IMP_ and the relative expression of *acrB* in these two strains were similar, but the *ompk36* expression level of KP-I2, membrane porin gene, were relatively lower, which may be the main reason for its higher resistance to meropenem.

**FIGURE 7:**
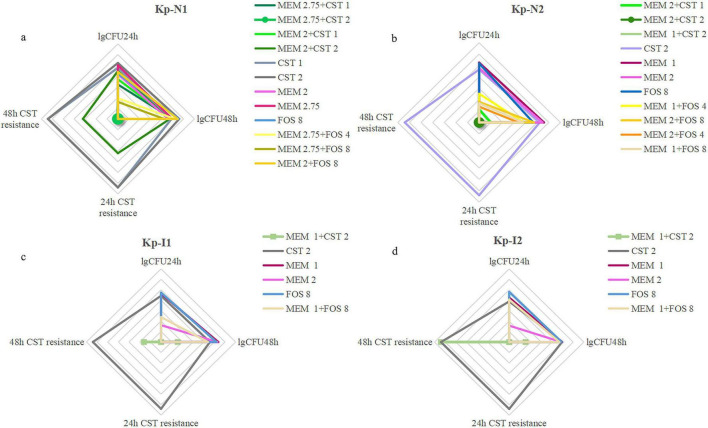
A comprehensive evaluation of bactericidal and drug resistance under all regimens of **(a)** Kp-N1, **(b)** Kp-N2, **(c)** Kp-I1, **(d)** Kp-I2. The four axes arranged radially in the radar image represent the degree of resistance that occurred at 24 h, 48 h, and colony counts from *in vitro* PK/PD model at 24 h, 48 h, respectively. The larger coverage area of the radar map represents a worse bactericidal efficacy and higher risk of colistin resistance. MEM 1, meropenem 1 g q8h; MEM 2, meropenem 2 g q8h; MEM 2.75, meropenem 2.75 g q6h; FOS 4, fosfomycin 4 g q8h; FOS 8, fosfomycin 8 g q8h; CST 1, colistin Css = 1 mg/L; CST 2, colistin Css = 2 mg/L.

Regarding drug screening and dose optimization, an expert review suggests previously that meropenem should be avoided if the strain is positive for carbapenemase, even if it is susceptible to meropenem (Pascale et al., 2019). Our research confirmed that meropenem-based combination therapy was still effective against MBL-KP. The dosage of combination regimens should be adjusted according to the different enzyme-producing types and MIC. Figure 7 summarizes the recommended radar chart of the combination regimens according to the effectiveness and heterogeneous resistance indicators. For NDM-KP, the appropriate combination regimens were 2.75 g q6h meropenem + 2 mg/L colistin, 2.75 g q6h meropenem + 4 g q8h or 8 g q8h fosfomycin for KP-N1 with highly resistance to meropenem. Although the guidelines do not recommend the use of meropenem-containing combination therapy for CRE when MIC is higher than 32 mg/L (Tamma et al., 2021), checkerboard screening results showed that only meropenem contained combination regimens showed synergism, while *in vitro* PK/PD model study demonstrated only the combination regimen with meropenem at 2.75 g q6h showed a bactericidal effect. For KP-N2 strains showing mild resistance to meropenem, all monotherapy regimens were invalid, and all combination regimens were effective. Adjustment of the relative dose of two components in the combination did not affect the bactericidal curve of KP-N2. However, NDM-KP tended to occur heterogeneous drug resistance to colistin, which could be suppressed through increasing the meropenem dose and decreasing the colistin dose in a combination regimen. In addition, we found that although the initial colistin MIC of two NDM-KP strains was both 1 mg/L, there was little difference in the MIC of other drugs, and KP-N1 with higher meropenem MIC was more likely to produce heterogeneous resistance to colistin. Therefore, the MIC value of meropenem should be considered for drug options for NDM-KP. Meropenem in combination with fosfomycin or high-dose meropenem in combination with colistin was suitable for NDM-KP extremely resistant to meropenem due to the lower heterogeneous risk of colistin. For IMP-KP, our *in vitro* study showed that the effect of high-dose meropenem (2 g q8h) monotherapy was similar to that of low-dose meropenem (1 g q8h) combination regimen. Compared with NDM-KP, IMP-KP was more easily to have heterogeneous resistance in all colistin-containing regimens, including the combination regimen with the excellent bactericidal effect. Although our study suggested that the heterogeneous resistance could be reduced through increasing meropenem dose and decreasing colistin dose, high-dose monotherapy of meropenem or in combination with fosfomycin was still the first recommended choice for IMP-KP.

The murine model of peritoneal sepsis was selected because KP is responsible for inducing peritonitis and shows similar hemodynamic and physiological changes to those in human sepsis (Deitch, 2005, Murando et al., 2019). The death usually occurs within 48 h, and the mortality rate can be controlled by modifying the dose of KP. In general, *in vivo* animal experiments preliminarily confirmed the effectiveness of the meropenem-based combinations in MBL-KP infections, which could reduce the heterogeneous drug resistance to colistin.

However, there were still limitations to the research. The quantity of strains utilized is comparatively small, and owing to the intricacy of the human internal environment and the substantial discrepancies between humans and animals, the murine model of peritoneal sepsis is unable to entirely replicate the process of bacterial infections and drug dosage optimization in humans. Thus, large real-world research evidence is still needed to verified the current findings.

## 5 Conclusion

Collectively, meropenem in combination with fosfomycin or colistin was an effective choice for MBL-KP, after the targeted combination regimen selection and dose optimization according to the enzyme type and resistance phenotype. The rationalized application might be a more practical way to realize the current carbapenem-sparing policy.

## Data Availability

The datasets presented in this study can be found in online repositories. The names of the repository/repositories and accession number(s) can be found in the article/Supplementary material.

## Footnotes

1
http://www.genomicepidemiology.org

2
http://cge.cbs.dcu.dk/services/ResFinder/
