# Supplementary material for: Carbapenem treatment options for metallo-beta-lactamase: drug screening and dose optimization of meropenem-based combinations against NDM- or IMP-producing Klebsiella pneumoniae
Source: Front Microbiol. 2025 Apr 15;16:1490372. doi: 10.3389/fmicb.2025.1490372 (PMC12037592; doi:10.3389/fmicb.2025.1490372)
Supplement: Supplementary file 1 [file Table_1.doc]

**Bacterial isolates**

Four clinical KP isolated strains were stored at -80°C in brain heart infusion broth (AOBOX, Beijing, China) and passaged twice on 5% sheep blood agar before further analyses. Fresh colonies were inoculated onto Mueller-Hinton Agar (MHA) (AOBOX, Beijing, China) plates each week for all experiments. Information of patients and antimicrobial resistance of four isolated strains were listed in table 1 (Supplementary Table 1).

**Synergy testing and target drug regimen screening**

The synergistic activity of meropenem in combination with other antibiotics was assessed by the checkerboard method. All Mueller-Hinton Broth (MHB)-involving fosfomycin were adjusted with glucose 6-phosphate (G6P) (25 mg/L). *Escherichia coli* ATCC25922 was used in this study as a quality control strain. The fractional inhibitory concentration index (FICI) was calculated using the formula as follows:

FICI =FICI_A_ + FICI_B_,

where FICI_A_ is MIC of drug A in combination/MIC of drug A alone, and FICI_B_ is MIC of drug B in combination/MIC of drug B alone.

**Real-time reverse-transcription polymerase chain reaction**

Total RNA was extracted using the RNeasy-kit (TIANGEN®, Beijing, China), and purified RNA was reversely transcribed using the cDNA synthesis kit (Thermo Fisher, Massachusetts, USA). RT-PCR was then performed using a CFX96 Touch RT-PCR Detection System (Bio-Rad, California, USA) with the TB Green® *Premix Ex Taq*™ RT-PCR Kit (TaKaRa, Tokyo, Japan) according to the manufacturer’s instructions.

The *rpoB* gene was selected as the housekeeping gene, the KP ATCC700603 was used as the reference strain for *acrB*, and the KP ATCC13883 was used as the reference strain for *ompk35* and *ompk36* (expression level = 1). The relative expressions of the target genes were calculated using the 2^-ΔΔCT^ method. All primer sequences were listed in table 2 (Supplementary Table 2).

**Validation of *in vitro* PK/PD model**

To verify the accuracy of the model, the concentrations of meropenem in the central compartment were measured by liquid chromatography/tandem mass spectrometry (LC-MS/MS) with the AJS/ESI mode. The sample was pretreated by filtration. The precursor to product ion transitions monitored for meropenem and internal standard (fluconazole) were m/z 384.0→142.0 and 307.0→219.9, respectively. The lower limit of quantification (LLOD) for meropenem was 0.1 mg/L. For drug concentration in the *in vitro* PK/PD model, a linear regression was performed, and the global fit was assessed with the coefficient of determination (R2), which represented the proportion of the variability of the dependent variable (Y) that could be attributed to X.

Supplementary Fig. 2a shows the measured concentrations for meropenem over 24 h of the *in vitro* PK/PD model, which were basically consistent with the target concentration curve. Overall, the measured versus target drug exposures of meropenem achieved in this model were considered satisfactory based on observed R^2^ values of 0.82 (Supplementary Fig. 2b).

**Experimental murine model peritoneal sepsis**

C57BL/6J female mice (20 g, 8 weeks old) were used (SiPeiFu, Beijing, China) in the present study, which were infected with each strain. The bacterial suspension of approximately 2 McFarland (6 × 10^8^ CFU/mL) in MHB were made for inoculation. Septicemia was induced by intraperitoneal injection (IP) of 0.5 mL of the inoculum at 2 h before the initiation of antimicrobial therapy.

**Efficacy studies**

Infected mice of each strain were randomly divided into 1 controls groups (normal saline), 4 monotherapy groups(low dose meropenem, high dose meropenem, colistin, fosfomycin), and 4 combination groups(low dose meropenem combined with colisin, high dose meropenem combined with colistin, low dose meropenem combined with fosfomycin, high dose meropenem fosfomycin), with 6 mice per-group. Treatments were initiated at 2 h post-inoculation and lasted for 24 h. Administration time, dosage, and route of each antibiotic were shown in Table 4 (Supplementary Table 4).

The survived mice were sacrificed through inhalation of isoflurane (Veteasy, Shenzhen, China) at 24 h. Samples were extracted and processed immediately. At 24 h, the blood sample was taken through eyeball extraction, aseptic thoracotomies were carried out in all experimental mice, spleen samples were aseptically extracted, weighed, and homogenized in sterile saline, and quantitative cultures were determined by MHA plates. In the experiment containing colistin, all samples were additionally coated with four times colistin at the MIC concentration for quantification to verify the formation of heterogeneous drug resistance in animals.

This part of the study was carried out following the recommendations of the Guide for the Care and Use of Laboratory Animals and modified based on a previously published study(Cebrero-Cangueiro et al., 2021). *In vivo* experiments were approved by the Institutional Animal Care and Use Committee of the Chinese PLA General Hospital (2020-X13-71).

**
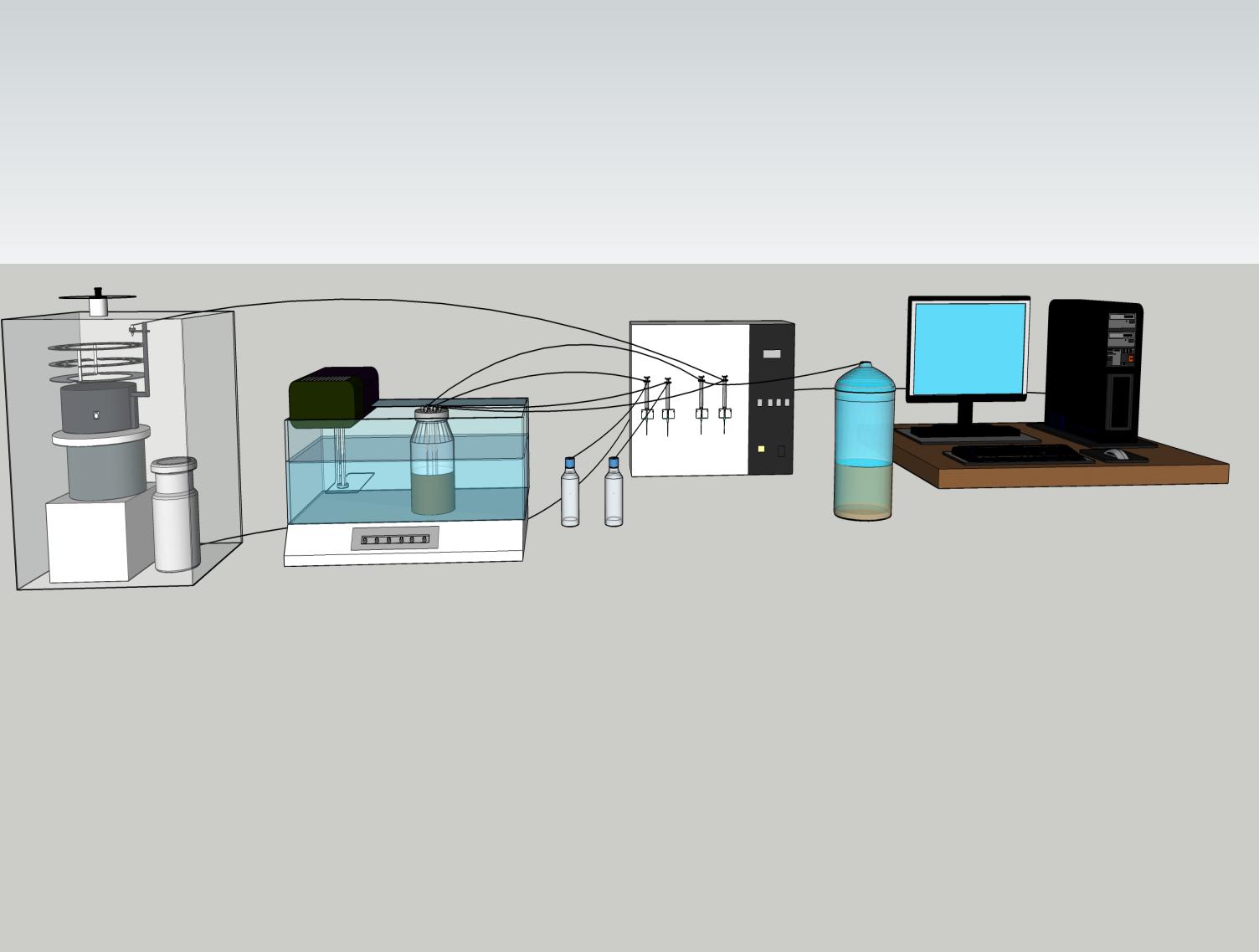
**

**Supplementary Figure** **1.** Schematics of the *in vitro* PK/PD model.


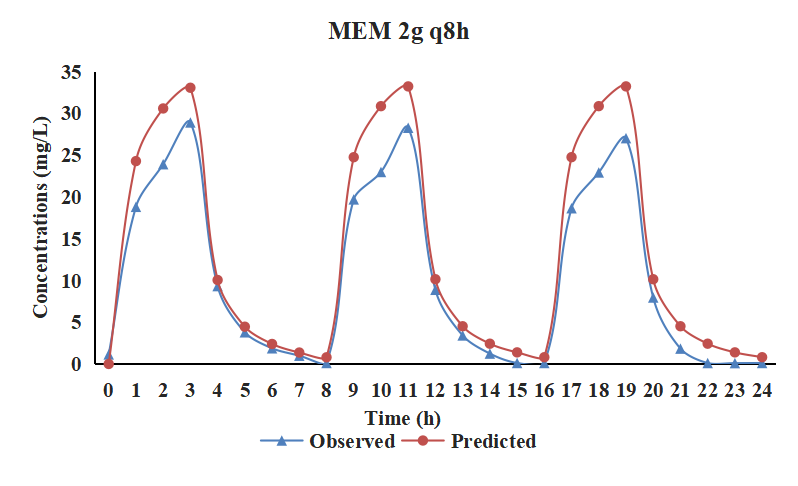

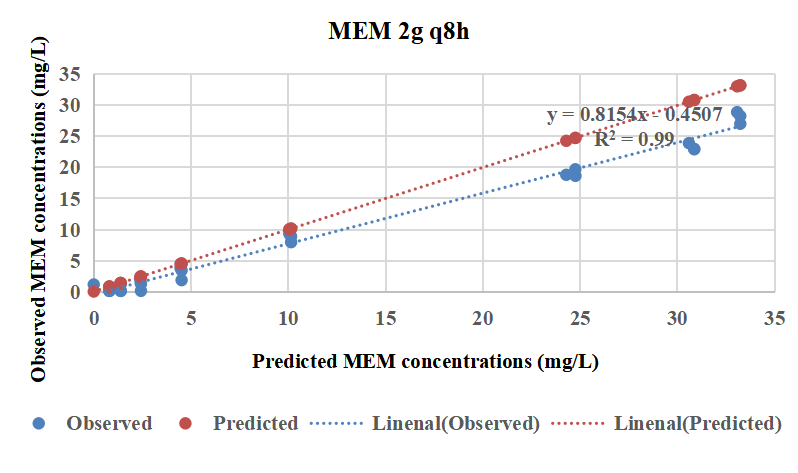


**a**

**b**

**Supplementary** **Figure 2. (a)** Observed and predicted concentration-time curves of meropenem 2 g q8h from *in vitro* PK/PD model. **(b)** Relationships between observed and predicted meropenem concentrations. MEM: meropenem.

**Supplementary Table 1.** Information of patients and antimicrobial resistance of four isolated strains.

| Strains | Kp-N1 | Kp-N2 | Kp-I1 | Kp-I2 |
| --- | --- | --- | --- | --- |
| Information of patients | | | | |
| Gender | Female | Male | Male | Male |
| Age(old) | 52 | 61 | 84 | 85 |
| Department | Extravascular | Oncology Department | Respiratory Department | Respiratory ICU |
| Specimen | Urine | Drainage | Sputum | Urine |
| Antimicrobial therapy before sampling | None | Ceftriaxone Sodium and Sulbactam Sodium | Meropenem, Fluconazole, Linezolid, Piperacillin Sodium and Tazobactam Sodium | Cefoperazone Sodium and Sulbactam Sodium, Minocycline, Fluconazole, Meropenem |
| Drug resistance information of clinical test report | | | | |
| ESBL | Negative | Negative | Negative | Negative |
| Ampicillin | R | R |  | R |
| Ampicillin/Sulbactam |  |  | R |  |
| Piperacillin/Tazobactam |  |  | I |  |
| Piperacillin | R | R | R | R |
| Cefazolin | R | R | R | R |
| Cefotetan |  |  |  |  |
| Ceftazidime | R | R | R | R |
| Cefoperazone/Sulbactam |  |  |  |  |
| Cefatriaxone | R | R |  | R |
| Cefepime | I | R | I | R |
| Cefuroxime |  |  | R |  |
| Cefotaxime |  |  | R |  |
| Aztreonam | R | I |  | R |
| Ertapenem | R | R | R | R |
| Imipenem | R | R | R | R |
| Amikacin | S | S | R | R |
| Gentamicin |  | S | R | R |
| Tobramycin |  |  |  |  |
| Ciprofloxacin |  | R | R | R |
| Levofloxacin |  | R | R | R |
| Nitrofurantoin | R | R |  | R |
| Compound Sulfamethoxazole | S | R | R | R |
| Amoxicillin / Clavulanic Acid | R | R | R | R |
| Meropenem | R | R | R | R |
| Tetracycline | R | R |  | R |

ESBL, extended-spectrum beta-lactamases; ICU, intensive care unit.

**Supplementary Table 2.** Primer sequences used for RT-PCR.

| Gene | Primers | Sequence 5'-3' | Reference |
| --- | --- | --- | --- |
| *rrsE* | *rrsE*-F | GTCATCATGGCCCTTACGAG | Yin Yuhan el |
|  | *rrsE*-R | ACTTTATGAGGTCCGCTTGCT |  |
| *ompk35* | *ompk35*-F | CAAAAACGGCAACAAACTGGACTTCT | Michel Doumith el |
|  | *ompk35*-R | TGGTCTGGGAACCTTCAACATTGGAC |  |
| *ompk36* | *ompk36*-F | AGGCGTGAAAGGCGAAACCCAG | Michel Doumith el |
|  | *ompk36*-R | AGGAAGTTCTCAGAACCGTAGG |  |
| *acrB* | *acrB*-F | AAACTTCGCCACTACGTCATA | Li Junjie el |
|  | *acrB*-R | AGCTTAACGCCTCGATCAT |  |
| *bla*_NDM_ | *bla*_NDM_-F | TGCGGCGCAACACAGCCTGA | [S Pollett](https://pubmed.ncbi.nlm.nih.gov/?term=Pollett+S&cauthor_id=25210072) el |
|  | *bla*_NDM_-R | TGGCCGGGGCCGGGGTAAAA |  |
| *bla*_IMP_ | *bla*_IMP_-F | ATTTTCATAGTGACAGCACGGGC | [S Pollett](https://pubmed.ncbi.nlm.nih.gov/?term=Pollett+S&cauthor_id=25210072) el |
|  | *bla*_IMP_-R | CCTTACCGTCTTTTTTAAGCAGCTCATTAG |  |
| *bla*_OXA_ | *bla*_OXA_-F | TGTTTTTGGTGGCATCGAT | Jussimara Monteiro el |
|  | *bla*_OXA_-R | GTAAMRATGCTTGGTTCGC |  |
| *bla*_KPC_ | *bla*_KPC_-F | GGCCGCCGTGCAATAC | [S Pollett](https://pubmed.ncbi.nlm.nih.gov/?term=Pollett+S&cauthor_id=25210072) el |
|  | *bla*_KPC_-R | GCCGCCCAACTCCTTCA |  |

**Supplementary Table 3**. PK parameters of meropenem and fosfomycin applied in the *in vitro* PK / PD model.

| Meropenem | | Fosfomycin | |
| --- | --- | --- | --- |
| K_10_ | 1.34 | T_1/2_ | 4h |
| V_c_ | 13.8L | K | 0.173286795 |
| V_p_ | 5.97L | V | 26.3L |
| K_12_ | 0.323 |  |  |
| K_21_ | 0.75 |  |  |

Meropenem was simulated with a two-compartment model, fosfomycin was simulated with an one-compartment model.

**Supplementary Table 4.** Administration scheme for *in vivo* animal experiments

| Antimicrobial agents | Dosage regimen | Route of administration |
| --- | --- | --- |
| MEM_low_ | 75mg/kg, 4 times/day | Intraperitoneal injection |
| MEM_high_ | 150mg/kg, 4 times/day | Intraperitoneal injection |
| CST | 100mg/kg, 6 times/day | Intraperitoneal injection |
| FOS | 5mg/kg, 4 times/day | Subcutaneous injection |

*The antimicrobial dosages were selected based on the PK/PD data in previous studies(Lepak et al., 2017, Oshima et al., 2017, Papp-Wallace et al., 2019).

**Reference:**
